# Proteorhodopsins dominate the expression of phototrophic mechanisms in seasonal and dynamic marine picoplankton communities

**DOI:** 10.7717/peerj.5798

**Published:** 2018-10-23

**Authors:** Ella T. Sieradzki, Jed A. Fuhrman, Sara Rivero-Calle, Laura Gómez-Consarnau

**Affiliations:** Department of Biological Sciences, University of Southern California, Los Angeles, CA, United States of America

**Keywords:** Phototrophy, Proteorhodopsin, Metatranscriptome, Metagenome, Spectral tuning, Marine, Picoplankton, Expression

## Abstract

The most abundant and ubiquitous microbes in the surface ocean use light as an energy source, capturing it via complex chlorophyll-based photosystems or simple retinal-based rhodopsins. Studies in various ocean regimes compared the abundance of these mechanisms, but few investigated their expression. Here we present the first full seasonal study of abundance and expression of light-harvesting mechanisms (proteorhodopsin, PR; aerobic anoxygenic photosynthesis, AAnP; and oxygenic photosynthesis, PSI) from deep-sequenced metagenomes and metatranscriptomes of marine picoplankton (<1 µm) at three coastal stations of the San Pedro Channel in the Pacific Ocean. We show that, regardless of season or sampling location, the most common phototrophic mechanism in metagenomes of this dynamic region was PR (present in 65–104% of the genomes as estimated by single-copy recA), followed by PSI (5–104%) and AAnP (5–32%). Furthermore, the normalized expression (RNA to DNA ratio) of PR genes was higher than that of oxygenic photosynthesis (average ± standard deviation 26.2 ± 8.4 vs. 11 ± 9.7), and the expression of the AAnP marker gene was significantly lower than both mechanisms (0.013 ± 0.02). We demonstrate that PR expression was dominated by the SAR11-cluster year-round, followed by other Alphaproteobacteria, unknown-environmental clusters and Gammaproteobacteria. This highly dynamic system further allowed us to identify a trend for PR spectral tuning, in which blue-absorbing PR genes dominate in areas with low chlorophyll-*a* concentrations (<0.25 µgL^−1^). This suggests that PR phototrophy is not an accessory function but instead a central mechanism that can regulate photoheterotrophic population dynamics.

## Introduction

Sunlight is the most readily available source of energy in the photic zone of the ocean. Light utilization in marine microorganisms is divided between complex, high-yield photosystems (oxygenic and anoxygenic photosynthesis) and simple, low-yield rhodopsins (Finkel, Béjà & Belkin, 2013). Light-harvesting mechanisms span the entire visible light spectrum, with bacteriochlorophyll-a and chlorophyll-*a* (Chl-*a*) utilizing its extremes, and various types of rhodopsins absorbing intermediate frequencies (Fuhrman, Schwalbach & Stingl, 2008). Since the discovery of proteorhodopsin proteins (Béjà et al., 2000; Béjà et al., 2001) they were found to be globally abundant across various oceanic regimes (Rusch et al., 2007; Boeuf et al., 2016; Brindefalk et al., 2016; Dubinsky et al., 2017). Among the known microbial rhodopsins, type-1 proton pumping proteorhodopsins (PR) are the most abundant and widespread in marine systems (Pinhassi et al., 2016); herein we will refer to all microbial rhodopsins as PR for simplicity. Genomic studies showed that the gene coding for PR is present in some of the most abundant bacteria in the ocean, e.g., SAR11 and SAR86 (Béjà et al., 2000; Sabehi et al., 2004; Giovannoni et al., 2005). PR-coding genes have also been found in some microbial eukaryotes such as fungi and photosynthetic protists, as well as in archaea and even in viruses as an auxiliary metabolic gene (Philosof & Béjà, 2013; reviewed by Pinhassi et al., 2016). PRs are the simplest light-harvesting mechanisms known to date, containing only one membrane protein and a retinal chromophore (Béjà et al., 2000). Light-driven proton pump PRs can increase the membrane potential of the cell, ultimately supporting a variety of processes such as ATP synthesis (Béjà et al., 2000; Walter et al., 2007; Steindler et al., 2011), substrate uptake (Steindler et al., 2011; Gómez-Pereira et al., 2013; Gómez-Consarnau et al., 2016), survival during starvation (Gómez-Consarnau et al., 2010; Steindler et al., 2011) and/or salinity stress response (Feng et al., 2013). Taken together, their structural simplicity and the range of functions they can support seem to have promoted the expansion of PRs in the sunlit ocean. However, estimates of the relative abundance of PR genes using metagenomics (Finkel, Béjà & Belkin, 2013; Brindefalk et al., 2016; Dubinsky et al., 2017) or metatranscriptomics (Shi et al., 2011; Kopf et al., 2015) have only been examined recently. In contrast to qPCR methods, next generation sequencing techniques can provide more reliable estimates without introducing qPCR and cloning biases that would miss certain PR gene types (Nguyen et al., 2015; Boeuf et al., 2016).

PR genes are highly expressed in the photic zone (Frias-Lopez et al., 2008; Poretsky et al., 2009; Satinsky et al., 2014). While generally transcription is not always an indicator of protein activity, one study shows good correlation between PR transcription and synthesis in a diatom (Marchetti et al., 2015) and another shows diel oscillations in PR transcription that peak before dawn, implying a preparation for light harvesting during the day (Ottesen et al., 2014). Bacteria in the genus Dokdonia upregulate the expression of PR genes in the light only in oligotrophic seawater conditions (Riedel et al., 2010; Gómez-Consarnau et al., 2016). Combined, these results may indicate that transcription levels are a good proxy for PR synthesis at least in some microbes. However, a cultured SAR11 strain has little regulation at the protein expression level and its PR genes appear to be constitutively expressed in light and dark (Giovannoni et al., 2005). All in all, there is limited information on the expression of PR compared to other light-harvesting mechanisms. In contrast with the established global distribution and abundance of PR taxonomic clusters, very few studies have compared their expression in environmental samples (Shi et al., 2011; Kopf et al., 2015; Boeuf et al., 2016; Brindefalk et al., 2016; Vader et al., 2018). Additionally, the vast majority of studies have been based on single time-points, with the exception of Sabehi et al. (2007), which compared winter and summer expression at two sites (Mediterranean and Sargasso Sea) and Nguyen et al. (2015), which compared early- and late-winter expression in the Arctic. Thus, information on temporal expression patterns of different PR clades remains scarce.

PR has two main variants that differ in their light absorption spectrum (Béjà et al., 2001; Man et al., 2003). This spectral tuning is determined by a single residue at the frequency-tuning site (FTS) (Man et al., 2003). It has been proposed that spectral tuning is related to the spectral quality and quantity of light in the water, i.e., water color. Consistent with this pattern, green-tuned PR are generally common in coastal waters, whereas the blue-tuned counterparts are typical of open-ocean or deeper water (Fuhrman, Schwalbach & Stingl, 2008) and references therein; (Pinhassi et al., 2016). For instance, while more than 70% of the PR sequences retrieved from the ultraoligotrophic Eastern Mediterranean were classified as blue-absorbing (Dubinsky et al., 2017), less than 10% belonged to this group in the eutrophic Baltic sea (Brindefalk et al., 2016). These data suggest that seasonal and more contrasting spatial variability could potentially determine the PR spectral tuning trends, namely that there is a direct correlation between spectral tuning and the trophic state of the water. However, no study to date has evaluated: (1) whether this distribution pattern actually applies to more dynamic environments with contrasting trophic conditions associated to seasonal and spatial gradients, or (2) whether the specific underwater light field could be an important ecological driver for photoheterotrophic populations in nutrient dynamic regions.

**Figure 1:**
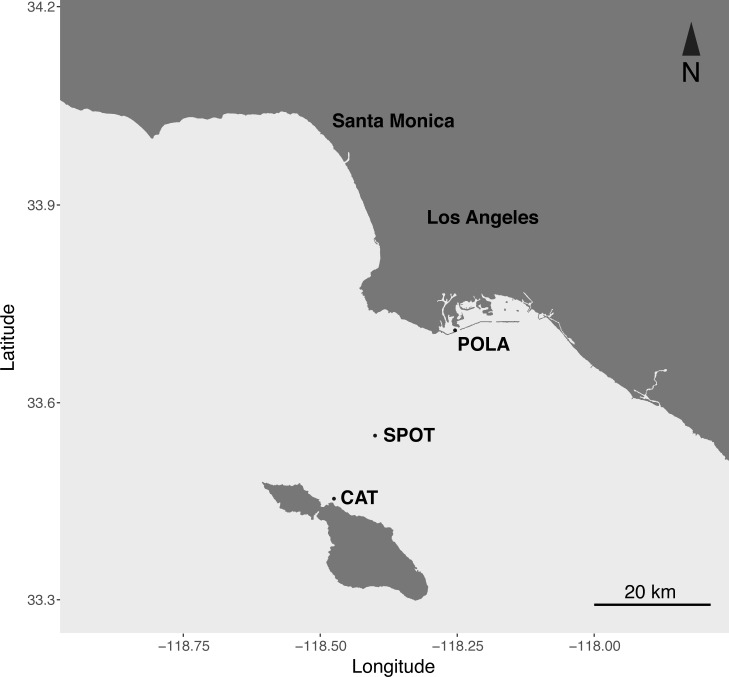
Map of the sampling sites. Port of Los Angeles (POLA, 33°42.75′N, 118°15.55′W), San Pedro Ocean Time-series (SPOT, 33°33′N, 118°24′W) and Catalina Island (CAT, 33°27.17 N, 118°28.51′W).

Here we present the first seasonal study of PR in metagenomes and metatranscriptomes of surface water microbial communities at three contrasting locations in the San Pedro Channel (Fig. 1). The transect spanned 37 km between the highly polluted Port of Los Angeles and the mildly impacted Santa Catalina Island, with the largely oligotrophic San Pedro Ocean Time-series (SPOT) halfway between them. The contrasting nature of those stations was observed in their inorganic nutrient concentration, chlorophyll levels, bacterial and viral counts and heterotrophic production (Table S1). We further compared PR abundance and distribution to the other two main phototrophic metabolisms in surface waters: oxygenic photosynthesis (PSI) and aerobic anoxygenic phototrophy (AAnP). Our data show that PR is the dominant phototrophic metabolism in microbial metagenomes and metatranscriptomes of this dynamic environment year-round.

## Material and Methods

### Sample collection

Ten liters of surface seawater (mixed layer depth 5–40 m) were collected from the Port of Los Angeles (POLA, 33°42.75′N 118°15.55′W), the San Pedro Ocean Time-series (SPOT, 33° 33.00′N 118°24.01′W) and Two Harbors, Santa Catalina Island (CAT, 33°27.18′N 118°28.51′W) in four seasons: July 2012, October 2012, January 2013 and April 2013. All samples were collected in the morning between 7 am and 12 pm. For nucleic acids extraction, seawater was prefiltered through a 1 µm glass fiber syringe filter (Pall, Acrodisc 25 mm) followed by a 0.22 µm Sterivex polyethersulfone filter (PES, Millipore, SVGPL10RC) with a maximum filtration time of 20 min. Due to high sequencing costs, no replicate samples were taken for metagenome and metatranscriptome analysis. 1.5 ml RNAlater was added into the Sterivex filters and they were sealed, flash frozen in liquid nitrogen and stored at −80 °C until extraction.

### Chemical and biological parameters

Whole seawater for nutrients measurement were collected in triplicates in 50 mL conical tubes and kept in −20° C until flow-injection analysis at the Marine Sciences Institute Analytical Lab at University of California, Santa Barbara (http://www.msi.ucsb.edu/services/analytical-lab). Bacteria and viruses per ml seawater were counted on duplicate slides using SYBR green epifluorescence microscopy (Noble & Fuhrman, 1998; Patel et al., 2007). For chlorophyll-*a* measurement, triplicates of 50–500 mL of whole seawater were filtered onto 25 mm GF/F filters and stored at −20 °C until analysis within the week. Filters were extracted with 4 mL of 100% acetone at −20 °C overnight in the dark, and processed on a calibrated Trilogy Laboratory Fluorometer (Turner Designs, San Jose, CA, USA) using the non-acidification method (Welschmeyer & Naughton, 1994). See Table S1 for all measured values.

### Nucleic acids extraction and Library preparation

After thawing the Sterivex filters, RNAlater was removed using a syringe in order to improve DNA yield. Cells on the filters were lysed by bead-beating for two cycles of 10 min each with 0.1 mm glass beads in 1.5 ml Sodium chloride-Tris-EDTA (STE) buffer injected into the Sterivex. DNA and RNA were then extracted from the flow-through using the AllPrep kit (Qiagen) that yields RNA and DNA from the same sample simultaneously. This process included treating the RNA columns with DNAse. The mean extraction yield was 2 µg DNA and 9 µg RNA per 10 L water. After extraction and quality assessment with Qubit HS (Thermo-Fisher Scientific, Waltham, MA, USA) and Bioanalyzer 2100 (Agilent, Santa Clara, CA, USA) nucleic acids were stored at −80 °C until further processing. As a sanity check, we used the reciprocal Qubit kits to quantify RNA in the eluted DNA and DNA in the eluted RNA and both were below detection in all samples. RNA samples were spiked with an internal standard for quantitative assessment of the sequencing process (ERCC RNA Spike-In Mix, Thermo Fisher 4456740; Thermo Fisher Scientific, Waltham, MA, USA) and converted to cDNA using the SuperScript III reverse transcriptase kit (Thermo Fisher Scientific, Waltham, MA, USA). Libraries were prepared using Ovation Ultralow library system V2 (0344; Nugen, Redwood City, CA, USA) from 200 ng DNA or cDNA mechanically sheared by Covaris M2. To ascertain that there were no contaminants in any reagents used in the extraction, library prep and sequencing processes, negative control libraries were generated by running Tris-EDTA buffer through the AllPrep kit and using the eluent as input for the Ovation ultralow library kit. All libraries were then sequenced on Illumina HiSeq 2 ×125 bp or 2 ×150 bp for metagenomes and 2 ×250 bp for metatranscriptomes.

### 16S/18S-rRNA amplification and sequencing

Hypervariable regions V4–V5 were amplified from DNA and cDNA of all samples following the protocol described in Parada, Needham & Fuhrman (2016) using dual barcoded primers. All PCR products were bead-cleaned with Ampure beads at a 1×beads to sample ratio, diluted to 1 ng/µl and pooled. The pool was bead cleaned again at a 0.8×beads to pool ratio. Insert size was verified on an Agilent 2100 Bioanayzer and the pool was sequenced on Illumina MiSeq 300 bp paired-end at the UC Davis genome core.

The resulting reads were quality-trimmed using Trimmomatic version 0.33 (Bolger, Lohse & Usadel, 2014) with parameters set to Leading:20 Trailing:20 Slidingwindow:15:25. The reads were then merged with Usearch 7 (Edgar, 2010) and analyzed using Mothur (Kozich et al., 2013) following the Miseq SOP (https://www.mothur.org/wiki/MiSeq_SOP) with an OTU preclustering cutoff of 2 mismatches and clustering at 99% sequence identity.

Reads that failed to merge, and were therefore more likely to represent 18S-rRNA, were first concatenated with an additional N base between the forward and reverse-complemented reverse read as described in (Needham & Fuhrman, 2016), and then clustered with usearch 6.1 (Edgar, 2010) at 97% identity via the Qiime 1 platform (Caporaso et al., 2010). Taxonomy was assigned using SILVA release 132 (Yilmaz et al., 2013). These reads were used only to estimate the relative abundance of diatoms in all samples.

### Metagenomes and metatranscriptomes sequence quality trimming

Quality trimming was performed using Trimmomatic 0.33 (Bolger, Lohse & Usadel, 2014) with parameters set to Leading:20 Trailing:20 Slidingwindow:15:25. Internal standard reads were removed from the metatranscriptomes informatically after confirming that their relative abundance out of all reads matched the relative abundance of the original spike-in. Metatranscriptomic reads were merged with PEAR (Zhang et al., 2013). Metagenomic reads could not be merged due to insert length and only the forward read was used for short read placement (see below). Sequencing depth after quality control is detailed in Table S2.

### Marker genes selection

The marker genes used for each of the light-harvesting mechanisms has previously been established (Finkel, Béjà & Belkin, 2013). Those are single-copy genes in most cases and can be found in all organisms that use the particular mechanisms. These genes can also be correlated to phylogeny, albeit not at very a high resolution and with the caveat that they (mainly PR) can be laterally transferred. Those marker genes can be used to track global distribution as well as expression. The PR gene codes for the transmembrane protein of proteorhodopsin, which anchors the retinal light-harvesting pigment. *psaA* codes for apoprotein a1 which binds P700, the main electron donor of photosystem-I, and *pufM* codes for chain M in the reaction center of the anoxygenic bacteriochlorophyll (Finkel, Béjà & Belkin, 2013).

### Assembly

Contigs were assembled within each metagenome/metatranscriptome separately with Megahit v1.0.4 (Li et al., 2015) and clustered with cd-hit (Fu et al., 2012) at 99% sequence identity to reduce complexity. Contigs longer than 2,000 kbp from all samples were then co-assembled with Minimus2 (Sommer et al., 2007) and shorter contigs were co-assembled with Newbler (Margulies et al., 2005). Both co-assemblies required a minimum overlap of 200 bp and a minimum sequence identity 99% and all resulting contigs were clustered again with cd-hit at 99% sequence identity. The overlap assembly was performed under the assumption that if longer contigs could have formed using kmer-based assemblers they would have done so within each sample.

### Marker gene extraction from assemblies

Open reading frames (ORFs) were identified in the assembled contigs using prodigal version 2.6.2 (Hyatt et al., 2010). The resulting translated ORFs were then scanned for PR, PsaA, PufM, PufL and RecA proteins via Anvi’o (Eren et al., 2015), and ORF sequences long enough to not affect the curated alignment (>200 aa, see below) were added to the protein dataset used for phylogenetic placement (see below). Assembled PsaA ORFs were all placed within the eukaryotic cluster possibly due to higher microdiversity of cyanobacteria. High microdiversity can cause assemblies to break (Martinez-Hernandez et al., 2017), explaining the lack of assembled cyanobacterial PsaA ORFs despite the fact that cyanobacterial PsaA was much more abundant in the small size fraction compared to picoeukaryotic PsaA (Fig. S1). Assembled PR ORFs represented multiple clusters and contributed significantly to recruitment (Table S3), and no assembled PufM ORFs matched our criteria. The gene *recA* was used to calculate the relative abundance of PR, PsaA and PufM in genomes (Brindefalk et al., 2016). Even though not all bacteria have *recA*, this gene has been previously assessed as nearly ubiquitous (Rocha, Santos & Pacheco, 2015) and is known to be present in all the taxonomic groups relevant in this surface marine environment. *recA* abundance was also previously shown to be very similar to other housekeeping genes (*gyrB*, *rpoB* and *tuf*) (Dubinsky et al., 2017).

### Phylogenetic trees

Curated protein subsets limited to aquatic bacteria, archaea, viruses and picoeukaryotes of PsaA, PufM and PufL were downloaded from Pfam (Finn et al., 2016) and RefSeq. These sets were supplemented by the respective assembled ORFs. Two sets of sequences were aligned using mafft (Katoh & Standley, 2013) (globalpair, gap open penalty 1.5, gap extension penalty 0.5 and scoring matrix BLOSUM30) and alignment trimming (Gblocks b3 = 50, b4 = 5, b5 = h, (Castresana, 2000): one set of *psaA* only and the other of *psbA*, *pufM* and *pufL* which are homologous. Each alignment was then used to build a Hidden Markov Model (HMM) using HMMER 3.0 (Johnson, Eddy & Portugaly, 2010) and a maximum likelihood tree with RAxML v8.2.5 (Stamatakis, 2014) using WAG substitution matrix and Gamma model (Ignacio-Espinoza & Sullivan, 2012). The trees are provided in Figs. S2 and S3.

A curated alignment and a phylogenetic tree of PR proteins was graciously provided by the MicRhoDE project (Boeuf et al., 2015) and included type-I proteorhodopsins as well as other rhodopsin clusters. The alignment was used to build an HMM of the PR amino acid sequence via hmmbuild. The assembled ORFs were first placed into the MicRhoDE tree (see Table S3 for placements) and the resulting tree was used for placement of short reads.

### Short reads placement

Most studies use either best blast hit or reciprocal blast to recruit reads to PR, whereas we used a combination of blastx, HMM (Hidden Markov Models) and placement of short translated reads into phylogenetic protein trees. This method almost always yielded many more reads than reciprocal blast, which is intentionally a very conservative estimate (Fig. S4).

All reads from the metagenomes and metatranscriptomes were searched against the curated protein datasets using blastx (Camacho et al., 2009) requiring an *e*-value of 10^−5^. Reads that hit those genes were translated and filtered again using the HMMs with hmmsearch. Hits with an *e*-value lower than 10^−5^ were aligned to the dataset using hmmalign. The aligned reads were placed into the phylogenetic trees with pplacer 1.1 run with default settings (Matsen, Kodner & Armbrust, 2010). Only reads that mapped to leaves (rather than internal nodes) were further analyzed.

The same process with the exception of placement into a phylogenetic tree was performed for RecA and used for normalization of the functional genes.

Gene abundances were determined by the formula used by Dubinsky et al. (2017)

\begin{eqnarray*}\text{(funcAbun/funcLen)/(RecAAbun/RecALen)} \end{eqnarray*}

where func is any functional gene (*psaA*, *PR* or *pufM*), funcAbun is the relative abundance of reads placed into leaves in the phylogenetic tree of this functional gene per sample, funcLen is the length of the HMM built for the functional gene, RecAAbun is the relative abundance of reads mapped to RecA per sample by HMM and RecALen is the length of the RecA HMM.

### Reciprocal blast

For comparability to previous papers, reads mapping to all genes were also extracted from the metagenomes and metatranscriptomes using reciprocal blast. First, we built a blast database from every metagenome and metatranscriptome. Then we used the curated sequences described above as a query to search these databases using tblastn (Camacho et al., 2009). The reads that resulted from this search were then searched against the NCBI non-redundant database (nr) using blastx (Camacho et al., 2009), and only reads that hit the desired genes were retained. General trends between genes were similar using this method compared to HMMs but the number of recruited reads was almost always significantly lower for the functional genes (Fig. S3).

### Spectral tuning of PR

Reads that mapped to the frequency tuning site (FTS) in the protein alignment (using HMMalign) were analyzed to determine tuning relative abundance of blue (glutamine) or green (leucine or methionine). While other residues were observed, they were extremely rare and therefore not included in the analysis.

### Underwater light field

Unfortunately, no in situ irradiance data could be collected at the time of sampling, therefore we resorted to well-established satellite remote sensing data. Ocean color satellites can return information on the underwater light quality and quantity through the remote sensing reflectance (Rrs, unit: sr^−1^) parameter, which is the relationship of upwelling water leaving radiance (Lw, units: W m^−2^ sr^−1^ nm^−1^) to downwelling irradiance (Ed, units: W m^−2^ nm^−1^) integrated over the first optical depth. Lw and Ed are the typical parameters that would have been measured in situ. Rrs is defined as:

\begin{eqnarray*}\text{Rrs}=\text{Lw/Ed}. \end{eqnarray*}

Since Rrs is wavelength-dependent, the shape and height of the spectrum can tell us about the availability and quality of the underwater light field.

### Satellite data acquisition and analysis

MODIS level 3 mapped daily 4 km resolution satellite products of remote sensing reflectance (Rrs) were downloaded from the NASA ocean color distribution website (https://oceancolor.gsfc.nasa.gov) in October 2017. Satellite products for this study were extracted for each location and date, spanning six days before and one day after sampling, thus exploring the short temporal variability as well. We averaged our satellite estimates within a 0.15 degree radius from each sampling location. With the exception of October, there was no satellite data on the exact day of sampling, therefore we used data from the next day.

### Statistics

Shannon index of evenness was calculated using the R package RAM. and one-sided paired *t*-test between gene abundance evenness and expression evenness was run using R basic package with mu = 0.

Spearman correlations were calculated using the corr.test function within the R package psych. This function can calculate Spearman correlations with *p*-value correction for multiple tests (we used option “fdr” for the correction).

### Map of sampling sites

The map was plotted using R package ggmap (Kahle & Wickham, 2013).

## Results

### Abundance and expression of light-harvesting mechanisms

**Figure 2:**
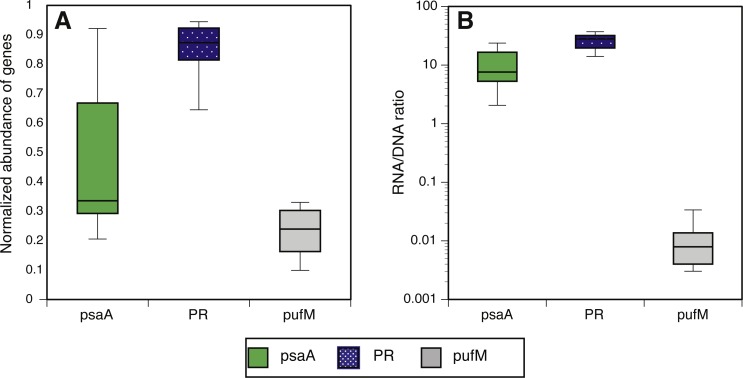
Normalized abundance and expression of phototrophic mechanisms. (A) Relative gene abundance of photosystem-I (PSI, *psaA*, green), rhodopsin (PR, dotted blue) and aerobic anoxygenic photosynthesis (AAnP, *pufM*, grey) normalized to *recA* in metagenomes and (B) RNA to DNA ratio per gene: the ratio between relative abundance out of all reads per sample in metatranscriptomes to relative abundance in metagenomes. Note that the *y*-axis in B is logarithmic.

We estimated the fraction of microbial cells that contain each of the light-harvesting mechanisms by normalizing the relative abundance of genes coding for microbial rhodopsins (*PR*), photosystem-I (*psaA*) and aerobic anoxygenic photosynthesis (*pufM*) to the relative abundance of the single-copy housekeeping gene *recA* (Finkel, Béjà & Belkin, 2013; Brindefalk et al., 2016; Dubinsky et al., 2017) which is also used as a baseline for RNAseq normalization (Rocha, Santos & Pacheco, 2015). The relative abundance of each of these genes in metatranscriptomes was then divided by their relative abundance in metagenomes to generate a normalized RNA to DNA ratio for each gene within each sample. Regardless of season or sampling location, the most common phototrophic mechanism in metagenomes was PR (65–104% compared to *recA*), followed by PSI (5–104%) and AAnP (5–32%) (Fig. 2A). In fact, in 10 out of 12 samples the *PR* gene exceeded 80% of *recA* abundance (Fig. S5). PSI normalized gene abundance was variable while PR remained within a narrower range through the different seasons and stations (Fig. 2A, Fig. S6). The expression of both *PR* and *psaA* was consistently 1–2 orders of magnitude higher than their respective gene abundance (Fig. 2B). The RNA to DNA ratio also revealed that while *pufM* gene abundance was sometimes comparable to *psaA* (Fig. 2A), its expression was 2–3 orders of magnitude lower (Fig. 2B). *pufM* expression was also 2–3 orders of magnitude lower than *pufM* gene abundance, suggesting that most of the AAnP bacteria in our samples were not actively performing this type of phototrophy at the time of sampling (Fig. 2B). Interestingly, we did not observe any geographical or seasonal trends for the presence of any light-harvesting strategies (Fig. 2).

**Figure 3:**
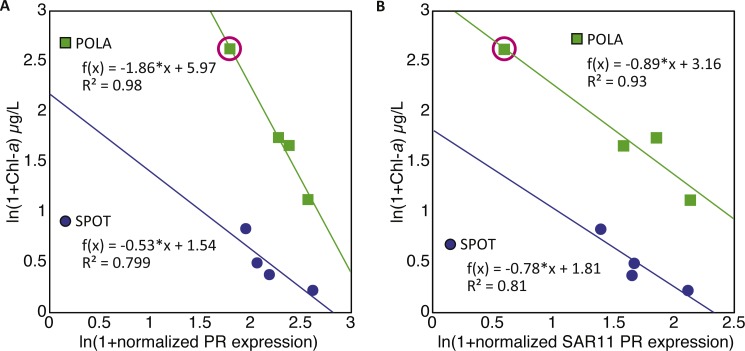
Negative correlation between proteorhodopsin (PR) expression and Chlorophyll—a at POLA and SPOT. (A) total PR expression (B) SAR11 PR-cluster expressionPR expression was normalized to *recA* expression. Trendline equations and *R*^2^ values are indicated on the plot. No correlation was found in CAT. The point representing POLA April 2013 is circled on the plots.

The combined normalized relative abundance of PR and PSI exceeded 100% in all samples analyzed, suggesting multiple gene copy numbers or coexistence of these mechanisms within the same prokaryotic cells (Finkel, Béjà & Belkin, 2013; Dubinsky et al., 2017), as previously shown in marine eukaryotic algae (Marchetti et al., 2012; Marchetti et al., 2015). While we did not observe any significant correlation between abundance of PR and PSI genes or transcripts, strong negative correlations between PR expression and total chlorophyll-*a* (Chl-*a*) concentrations were clearly identified at POLA and SPOT (Fig. 3A), and attributed to SAR11 PR expression (Fig. 3B). No significant correlation was found between PR expression and any other nutrient measured (Table S1). PSI gene abundance as well as expression were dominated by cyanobacterial genes rather than eukaryotic, despite the placement of assembled PsaA from contigs into the eukaryotic clade in the phylogenetic tree of this protein (Fig. S1, Table S3).

### PR distribution by cluster

**Figure 4:**
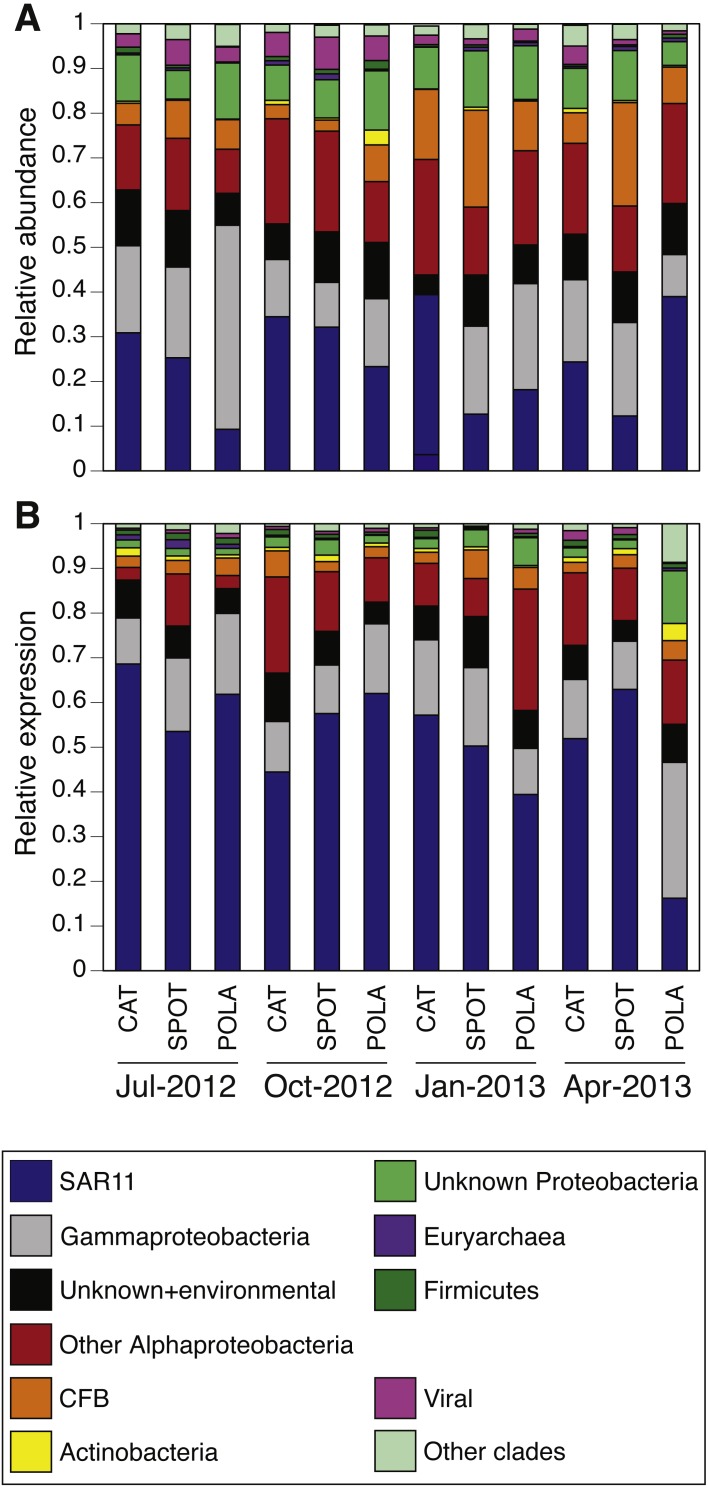
Relative abundance of various proteorhodopsins clusters (PR) out of all PR-assigned reads per sample. Relative abundance was calculated out of all rhodopsin-assigned reads per sample in (A) metagenomes (average *PR* reads per sample 6,485, standard deviation 3,927) and (B) metatranscriptomes (average *PR* reads per sample 43,005, standard deviation 22,367). The evenness of rhodopsin gene abundance was consistently high (see also sup. Fig. S7), whereas expression was dominated by SAR11 (dark blue), Gammaproteobacteria (grey), other Alphaproteobacteria (red) and unknown environmental clades 1 and 8 (black). The “Other clades” category includes rhodopsins of the MicRhoDE clusters Planctomycetes, bacteriorhodopsin, halorhodopsin, the “other rhodopsins” clade, NQ, xenorhodopsin, sensory rhodopsins, Betaproteobacteria, Deltaproteobacteria, Octadecabacter and Verrucomicrobia (Boeuf et al., 2015).

While our metagenomes revealed high taxonomic evenness of PR clusters (Fig. 4A, Fig. S7, average Shannon index of evenness 0.7 ± 0.06), expression was dominated by the SAR11 cluster (52 ± 14%) followed by Gammaproteobacteria (15 ± 6%), other Alphaproteobacteria (12 ± 7%) and unknown environmental clusters (8 ±2%) (Fig.  4B). Evenness in expression was always lower than evenness in gene abundance within a sample (one-sided paired *t*-test on Shannon index of evenness, *p* = 0.0003) except at POLA in April 2013 (Fig. S7). This particular sample was collected during a localized algal bloom with the highest Chl-*a* concentration measured in this study (12.7 µgL^−1^). The AAnP RNA to DNA ratio in this sample was the highest we detected, and this was the only time in which expression of PSI surpassed that of PR (Fig. S5). PR expression in this sample was dominated by Gammaproteobacteria (30%) and SAR11 PR expression dropped to 16%.

#### The SAR11 cluster

**Figure 5:**
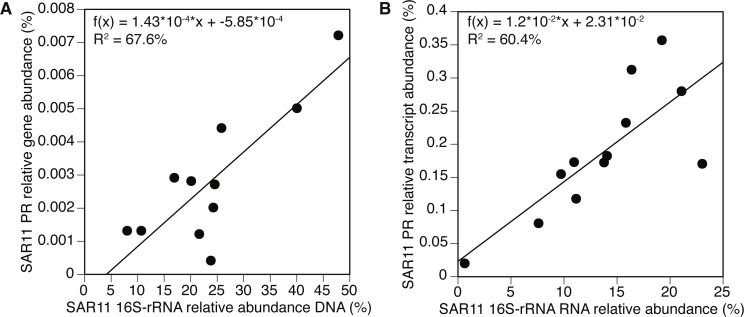
Positive correlation between 16S-rRNA and proteorhodopsin (PR) within the SAR11 clade. (A) Relative gene abundance and (B) relative transcript abundance. Sample collected at POLA in April 2013 was considered an outlier and therefore removed from the statistical analysis in (A).

**Figure 6:**
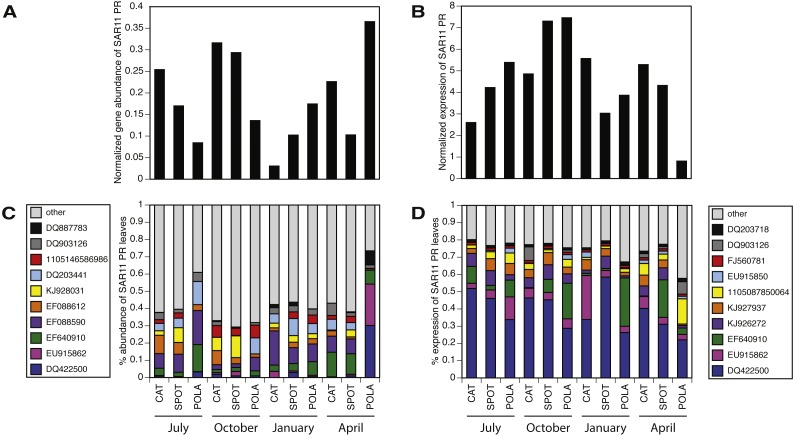
SAR11 cluster of proteorhodopsin (PR) at the single amino acid sequence level (per leaf in the MicRhoDE phylogenetic tree) had higher evenness in gene abundance than in expression (Wilcoxon rank sum test, paired one-sided, *p* = 0.0005). (A) SAR11 cluster PR total gene abundance over time and sites, (B) SAR11 cluster PR total expression over time and sites, (C) relative abundance of the 10 most abundant SAR11 cluster PR genes (all other SAR11 cluster PR were added up and represented as “other”, total 12,852 reads) and (D) relative abundance of the 10 most highly expressed SAR11 cluster PR (total 185,019 reads). Note that C and D have separate legends and that only 4 PR leaves are shared between them. Leaves are denoted by accession numbers.

We further examined the PR gene abundance and expression patterns of the SAR11 cluster, as this was the most abundant PR-containing group overall. SAR11 PR expression correlated positively with expression of SAR11 OTUs determined by 16S-rRNA (Fig. 5B). This correlation was also observed at the gene level after removing one outlier (POLA April 2013) (Fig. 5A). We further examined the presence and expression of specific SAR11 PR proteins within the SAR11 cluster by calculating read recruitment per leaf on the MicRhoDE phylogenetic tree of rhodopsins (Boeuf et al., 2015). We found that the high-resolution expression patterns were also much less even than gene distribution, where the top 10 most highly expressed leaves generally accounted for >70% of the SAR11 PR transcripts compared to less than 50% of gene abundance (Fig. 6). The mean Shannon index of evenness for gene abundance was 0.80 ± 0.09 (mean ± standard deviation), and significantly lower for expression: 0.57 ± 0.08 (Wilcoxon rank sum test, paired one-sided, *p* = 0.0005). Only four of the 10 most expressed SAR11 PR transcripts were also in the 10 most abundant SAR11 PR genes (Figs. 6C, 6D).

#### Other clusters

Although we found 13 viral PR open reading frames (ORFs) in our assemblies, the viral PR cluster did not appear to be expressed in this system (no more than 2.2% of the total PR transcripts per sample). Archaeal clusters were extremely rare (<1% of the metagenomic and <2% of metatranscriptomic reads recruiting to PR).

Expression and gene abundance of eukaryotic PRs were not apparent in any of the samples. Most of the eukaryotes that are known to carry PRs, such as diatoms and dinoflagellates, are large and not expected to be present in our <1 µm size-fraction (Marchetti et al., 2015; Vader et al., 2018). The picoeukaryotes *Micromonas* spp. and *Bathycoccus* spp. that have been shown to contain PR genes and are sometimes found in the San Pedro Channel were not present in our small size-fraction samples.

**Figure 7:**
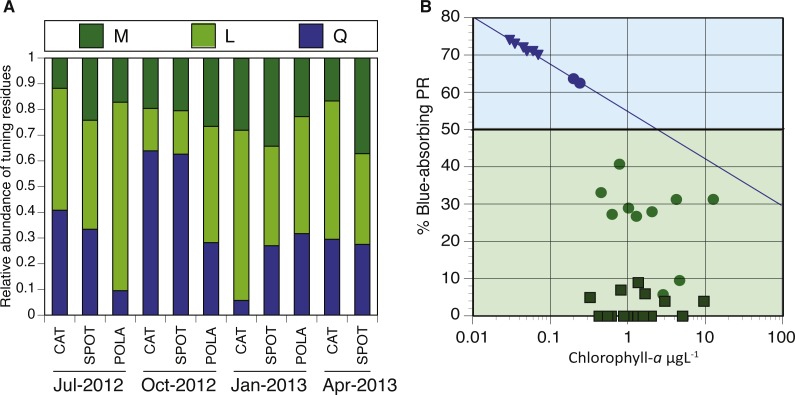
Spectral tuning of rhodopsins in our dynamic system and a comparison to previous studies. (A) Relative abundance of blue/green variants of proteorhodopsin (PR) in metagenomes (294–2,112 reads per sample): leucine (L) is represented by light green, methionine (M) by dark green and glutamine (Q) by blue. There was not enough data to plot tuning distribution at POLA in April 2013. (B) 0.25 µgL ^−1^ is the Chl-*a* threshold between environments dominated by blue-tuned PR or green-tuned based on data from this paper and previous publications. Triangles, squares and circles denote data from Dubinsky et al. (2017), Brindefalk et al. (2016) and this study, respectively. The trendline for samples with Chl-a concentration below 0.25 µgL^−1^ follows the equation %Blue- PR = [ − 0.055∗ln(Chl − *a*) + 0.55]∗100, *R*^2^ = 0.99.

### PR Spectral tuning

**Figure 8:**
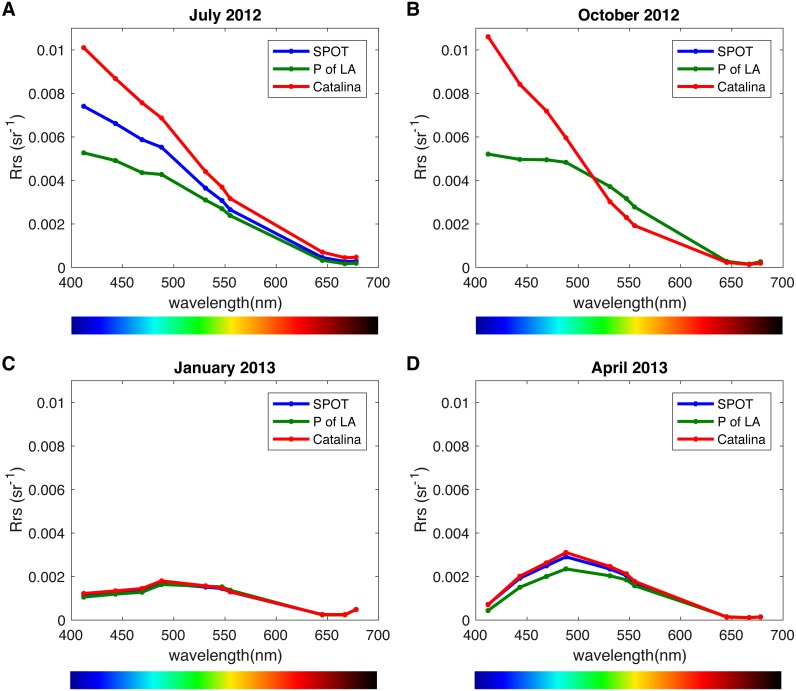
Remote sensing reflectance spectra (Rrs) per location and season. (A) July 16, 2012, (B) October 19, 2012, (C) January 10, 2012, (D) April 25, 2013. Measurements were taken the day after sampling day due to satellite data availability, with the exception of October for which there was data from the actual sampling day.

We analyzed the spatiotemporal distribution of the two main PR variants (blue and green) in the metagenomes and compared our results to previously reported data (Brindefalk et al., 2016; Dubinsky et al., 2017). Consistent with being a coastal environment, the majority of our samples were dominated by green-absorbing PR genes (Fig. 7A). However, samples collected at CAT and SPOT in October were dominated by the blue absorbing type, with 63% and 62% respectively. These two particular samples were collected on dates when Chl-*a* levels were the lowest measured in this study, below 0.25 µgL^−1^. Furthermore, the compilation of our data with values measured in the Eastern Mediterranean (Dubinsky et al., 2017) revealed a strong correlation between percent of blue-absorbing PR genes and the Chl-*a* concentrations only below 0.25 µgL^−1^ (Fig.  7B). However, the concentration of Chl-*a* in the water is only one of the components that determine water color. To fully evaluate the role of the underwater light field in the spectral tuning of PR, we compared the proportion of green and blue PR gene variants to the corresponding satellite products of remote sensing reflectance (Rrs). The satellite remote sensing reflectance data shows predominantly blue reflectance spectra for all locations in July and October and green spectra in January and April (Fig. 8). In the week preceding sampling in July Rrs spectra indicated an algal bloom that disappeared by sampling day and during which blue light availability decreased (Fig. S8).

## Discussion

### PR dominates gene abundance and expression of picoplankton light-harvesting mechanisms year-round

High abundance of PR genes was previously described in the Global Ocean Sampling data (Rusch et al., 2007) and in various other marine datasets (reviewed by Pinhassi et al., 2016; Brindefalk et al., 2016; Boeuf et al., 2016; Dubinsky et al., 2017; Maresca et al., 2018), and our observations further support this trend. However, most studies so far have focused on single time-points, and information on seasonal distribution of PR genes is lacking. Our experimental design allowed us to compare different contrasting locations and seasons, with the potential to identify patterns of phototrophy and resource availability. Previous studies in several marine environments (i.e., the North Atlantic and Arctic oceans as well as the Eastern Mediterranean Sea) found a correlation between PR gene abundance in genomes and Chl-*a* levels in seawater (Campbell et al., 2008; Boeuf et al., 2016; Dubinsky et al., 2017). Unexpectedly, we found that the presence of the PR gene in genomes was high year-round in this dynamic ecosystem, even at the eutrophic station of POLA, suggesting that the trophic state of the water is not always a good predictor of PR abundance.

PR expression in the San Pedro Channel was consistently higher than oxygenic photosynthesis in picoplankton over different seasons, with the exception of one sample taken during a localized algal bloom. A substantial amount of photosynthesis is performed by large photosynthetic eukaryotes and an important consideration is that AAnP bacteria and some cyanobacteria larger than 1 µm or particle-attached are excluded from our analysis. Nevertheless, picoplankton <1 µm can, in fact, represent the majority of the photosynthetic community at SPOT and CAT (Connell et al., 2017; Needham, Sachdeva & Fuhrman, 2017), and PR genes have been shown to be more abundant in this size fraction as well (Finkel, Béjà & Belkin, 2013). Our results support the previously reported low gene abundances of aerobic anoxygenic photosynthesis (AAnP) (Boeuf et al., 2013; Boeuf et al., 2016; Dubinsky et al., 2017) but we further show that its expression is significantly lower compared to the other light-harvesting mechanisms. These results need to be taken with a grain of salt however, since the expression of genes in the *puf* cluster happens mostly during the night (Koblížek et al., 2003; Wagner-Döbler & Biebl, 2006; Voget et al., 2015) and all our samples were collected in the early morning. Overall, we found no spatial trends in abundance or expression of PR genes, emphasizing the importance of this mechanism, but oxygenic photosynthesis and AAnP were significantly lower at POLA compared to the other stations. This highlights the importance of examining not just abundance of genes but also their expression when comparing these ubiquitous phototrophic strategies.

### PR expression is negatively correlated with chlorophyll-*a* concentrations

Despite the co-occurrence of PR genes and oxygenic photosynthesis *psaA* genes, PR gene expression appeared to be negatively correlated to Chl-*a* concentrations at SPOT and POLA. A similar trend was previously reported for gene abundance in the Arctic Ocean (Boeuf et al., 2016) and the Eastern Mediterranean (Dubinsky et al., 2017). Furthermore, this correlation was due to the expression trends of the SAR11 cluster of PR. We speculate that the slope of this correlation was steeper for total PR at POLA compared to SPOT due to the higher abundance of large photosynthetic eukaryotes at POLA (Connell et al., 2017), which can lead to more available organic carbon due to leaky cells and sloppy feeding. High availability of organic carbon enables cells to acquire energy heterotrophically rather than harvesting light, creating an environment in which the slow-growing oligotrophic-adapted SAR11 should be outcompeted (Giovannoni, 2017). To further support this, the slope of the correlation in SAR11 PR was similar between SPOT and POLA, implying that this clade is potentially not affected by high availability of labile carbon. Consistent with this hypothesis, in terms of physiology, PR phototrophy has been shown to be particularly important under DOM-limiting conditions typical of oligotrophic/low-chlorophyll regimes (Steindler et al., 2011; Gómez-Consarnau et al., 2007; Gómez-Consarnau et al., 2010; Gómez-Consarnau et al., 2016). This was especially clear at POLA in April 2013, when a localized diatom bloom was observed which did not extend to the other sites and led to a microbial eukaryotic community composition entirely divergent from all other samples (Hu et al., 2016; Connell et al., 2017). Notably, this was the only sample in which the expression of *psaA* genes for oxygenic photosynthesis exceeded that of PR genes. The high expression of *psaA* in this sample might be explained in part by the presence of chloroplasts released from diatoms that broke during the filtration and ended up being collected on the 0.2 µm filter. As the cumulative abundance of photosynthetic eukaryotes was significantly higher in this particular sample (Connell et al., 2017), and the relative abundance of diatom 18S DNA and RNA in it was higher than or equal to that of all other samples combined, this artifact is much more likely to have occurred in this sample. While the relative abundance of PR genes by clusters in this sample was similar to others, the expression pattern demonstrated an exchange of dominance between SAR11 and Gammaproteobacteria, most likely as a succession response to the algal bloom (Needham & Fuhrman, 2016).

### SAR11 is the most highly expressed PR cluster

Owing to the differences between the present PR-bearing community and its active subset, PR gene abundance distribution by cluster was more even at the gene level, as earlier observed in the Red Sea (Philosof & Béjà, 2013). The SAR11-cluster of proton-pump type PR dominated PR transcripts. Furthermore, the expression of this cluster correlated positively with the expression of SAR11 16S-rRNA operational taxonomic units (OTUs, 99% identity). This correlation was maintained at the gene level with the exception of POLA in April, potentially due to the interference introduced by the diatom bloom. These matching trends of presence and expression of the SAR11 PR aligns with the streamlined nature of SAR11 genomes and their reported constitutive expression of this protein (Giovannoni et al., 2005).

Other rhodopsins (e.g., actinorhodopsin, bacteriorhodopsin, xanthorhodopsin, halorhodopsin and xenorhodopsin) were rare. The abundance and expression of viral PRs was also very low, consistent with the fact that viral PRs were so far detected only in giant Phycodnaviruses of freshwater eukaryotes (Yutin & Koonin, 2012) and in low-salinity water (Brindefalk et al., 2016). While there is an inherent problem with normalizing both abundance and expression of viral PRs, as there are no universal viral marker genes to normalize to, this limitation was unlikely to affect our results due to the low relative abundance of viral PR. However, this should be taken into account in studies where the viral PR cluster is better represented.

### PR spectral tuning has a key role in population dynamics

Some studies show that blue light absorbing PR variants dominate in open ocean, oligotrophic conditions, whereas the green variants are more abundant in shallow or coastal water (Man et al., 2003; Rusch et al., 2007). However, these observations are not consistent in the literature, since the different PR types were also found to be decoupled from water parameters (Sabehi et al., 2007). As conditions at SPOT and CAT are dynamic and fluctuate seasonally between oligotrophic and mesotrophic conditions (Cram et al., 2015; Connell et al., 2017), we expected the spectral tuning of PRs to vary throughout the year. We observed a majority of blue absorbing PRs only in October of 2012, which coincided with the lowest Chl-*a* concentrations recorded in this study. Additionally, despite similar availability of blue light in July and October, PR tuning was dominated by the blue variant only in October. It is possible that the presence of an algal bloom during the week before sampling in July led to dominance of the green variant which the system did not recover from by sampling day. Comparing our results with published data from the ultraoligotrophic Eastern Mediterranean Sea (Chl-*a* < 0.01 µgL^−1^, (Dubinsky et al., 2017), we observed a Chl-*a* concentration pivot point of about 0.25 µgL^−1^. When Chl-*a* concentration was below this threshold, as in the Mediterranean and at SPOT and CAT in October, there was a highly significant negative correlation of chlorophyll concentration with PR expression, as well as high (60–75%) percent blue absorption at the gene level. However, above this threshold the percentage of cells with the blue variant PR genes dropped to an average of about 30% with no clear correlation to Chl-*a*. Similarly, Brindefalk et al. (2016) found even higher relative abundances of the green PR gene variant (>90%) in the Baltic Sea (Brindefalk et al., 2016) co-occurring with Chl-*a* levels significantly above 0.25 µgL^−1^. However, Chl-*a* concentration is only a proxy for the quality and quantity of light in the water column and the dissolved organic material (DOM) available. Under bloom conditions, Chl-*a* concentrations increase, turning the water greener and reducing its transparency. The consequent increase in available DOM that follows blooms further attenuates light in the water column, particularly in the blue and UV region of the spectrum. The resulting combination of reflectance and absorption by algal pigments, dissolved organic matter, water and inorganic particles is what determines the available light in the water column. When DOM is low, light harvesting and spectral tuning of PR may play a crucial role in survival or fitness of photoheterotrophic bacterial populations. This is evident in the most oligotrophic locations such as the Eastern Mediterranean (Dubinsky et al., 2017). Consistent with this, in year-round eutrophic locations such as the Baltic Sea, the dominant variant is green whereas in dynamic locations such as SPOT and POLA, a mix of the two variants can be expected. Since we can readily identify these patterns at the gene level, our data suggests that the light regime is a key factor driving selection in PR-containing populations, as suggested in the past (Sabehi et al., 2007). Since we did not collect in situ DOM or irradiance measurements, future studies will be needed to better define and increase the resolution of these thresholds to better understand the role of light quality and availability in population dynamics, survival and competition.

## Conclusion

Our spatial time-series analysis of PR and oxygenic and anoxygenic photosynthesis in marine picoplankton revealed that (1) expression of PR-based photoheterotrophy exceeded that of oxygenic photoautotrophy, (2) PR expression was dominated by few clusters despite a more even presence of diverse PR clusters, and (3) aerobic anoxygenic photosynthesis gene abundance appears to be relatively rare compared to the other mechanisms in this system. It is highly important to continue collecting more deeply-sequenced metatranscriptomic data in order to begin to elucidate the local adaptations of photoheterotrophs in the ocean that lead to their global success. Finally, our results reinforce the conclusion that the differences in the light spectrum are an important selective force, defining the abundance of different PR photoheterotrophic types.

##  Supplemental Information

10.7717/peerj.5798/supp-1 Table S1 Nutrients and Chlorophyll-A concentrations, cell and viral counts per ml and heterotrophic production measured in the contrasting sampling locations, San Pedro Channel All Chl-*a* measurements as well as nutrient measurements in October through April are courtesy of the Caron lab, USC. Values in bold are below the instrument detection limit.

10.7717/peerj.5798/supp-2 Table S2 Number of post quality control reads (sequencing depth) per sample in metagenomes and metatranscriptomes See data availability section for raw data accession numbers.

10.7717/peerj.5798/supp-3 Table S3 Placement of assembled proteorhodopsin ORFs in the MicRhoDE tree clusters indicates that locally assembled rhodopsins represent much of the PR diversity The number of reads (pooled from all samples) that mapped to the assembled ORFs from metatranscriptomes (MT) and metagenomes (MG) was significant compared to number of pooled reads mapped to the curated proteins from MicRhoDE.

10.7717/peerj.5798/supp-4 Figure S1 Read recruitment to *psaA* of cyanobacterial and eukaryotic origin demonstrated that the majority of *psaA* reads were prokaryotic in both metagenomes and metatranscriptomes (A) relative gene abundance of *psaA* by domain (B) relative transcript abundance of *psaA* by domain.

10.7717/peerj.5798/supp-5 Figure S2 Maximum-likelihood tree of PsaA protein sequences used to build the HMM and to recruit short reads with pplacer Bacterial sequences appear in blue, viral in red, eukaryotic in green and assembled ORFs in black.

10.7717/peerj.5798/supp-6 Figure S3 Maximum-likelihood tree of PufM and its homologue PsbA protein sequences used to build the HMM and to recruit short reads with pplacer *PufM* sequences appear in red and P*sbA* sequences in black.

10.7717/peerj.5798/supp-7 Figure S4 Comparison of relative abundance (number of reads recruited over total number of reads per sample) of functional genes using reads recruited by HMM or reciprocal blast (A) in metagenomes and (B) in metatranscriptomes. Note that the *Y*-axis in B is logarithmic.

10.7717/peerj.5798/supp-8 Figure S5 Gene abundance and RNA/DNA ratio of different phototrophic mechanisms by sample (A) Normalized gene abundance and (B) expression of oxygenic photosynthesis (psaA, green), rhodopsin (PR, dotted blue) and anoxygenic photosynthesis (AAnP, grey).

10.7717/peerj.5798/supp-9 Figure S6 Variability of phototrophic mechanisms in metagenomes from the three sampling locations of the San Pedro Channel ( *n* = 4, shown in Fig. 1) *Y*-axis denotes the percentage of genomes with the particular phototrophic mechanism normalized to *recA* gene (see methods). *psaA* for oxygenic photosynthesis, PR for proteorhodopsin and *pufM* for aerobic anoxygenic photosynthesis.

10.7717/peerj.5798/supp-10 Figure S7 Shannon index of evenness of rhodopsin clusters across samples Evenness in metagenomes is denoted by black bars, and in metatranscriptomes by striped bars.

10.7717/peerj.5798/supp-11 Figure S8 Remote-sensing reflectance spectra (Rrs) in the week preceding the July 2012 sampling shows recovery from an algal bloom which could explain the discrepancy in PR tuning between July and October

## Additional Information and Declarations

### Data Availability

The following information was supplied regarding data availability: All raw data (16S/18S, metagenomes and metatranscriptomes) can be found on EMBL-ENA under project number PRJEB12234. Metatranscriptomics sequences accession numbers are ERS1864892–ERS1864903, and negative control library sequences accession number is ERR2089009. Metagenomic sequences accession numbers are ERS1869885– ERS1869896 and negative control accession number is ERS1872073. Assembled amino acid sequences of Prd, PsaA and RecA can be found at Sieradzki, Ella (2018): Dimensions of Biodiversity—San Pedro Channel. figshare. Collection. https://doi.org/10.6084/m9.figshare.c.4099757.v1.
