# Supplementary material for: Proteorhodopsins dominate the expression of phototrophic mechanisms in seasonal and dynamic marine picoplankton communities
Source: PeerJ. 2018 Oct 23;6:e5798. doi: 10.7717/peerj.5798 (PMC6202958; doi:10.7717/peerj.5798)
Supplement: Table S1 — All Chl-a measurements as well as nutrient measurements in October through April are courtesy of the Caron lab, USC. Values in bold are below the instrument detection limit. [file peerj-06-5798-s001.docx]

| Date | Site | Phosphate (µM) | Silicate (µM) | Nitrite + nitrate (µM) | Chlorophyll-a (µg/L) |
| --- | --- | --- | --- | --- | --- |
| July 2012 | POLA | 0.20 | **0.56** | **0.19** | 4.67 |
|  | SPOT | 0.19 | 1.81 | **0.02** | 0.45 |
|  | CAT | 0.13 | 1.08 | **0.04** | 0.78 |
| October 2012 | POLA | 0.24 | 3.36 | 0.24 | 2.06 |
|  | SPOT | **0.09** | 1.46 | **0.15** | 0.24 |
|  | CAT | 0.22 | 1.66 | 0.24 | 0.20 |
| January 2013 | POLA | 0.34 | 2.45 | 2.14 | 4.24 |
|  | SPOT | 0.12 | **0.37** | 0.34 | 1.29 |
|  | CAT | 0.13 | **0.68** | 0.38 | 2.86 |
| April 2013 | POLA | 0.20 | 1.68 | **0.11** | 12.71 |
|  | SPOT | 0.22 | 1.80 | **0.14** | 0.63 |
|  | CAT | 0.13 | **0.62** | 0.26 | 1.02 |

| Date | Site | Bacteria/archaea (cells/ml) | Viruses (per ml) | Bacteria/archaea standard deviation | Viruses standard deviation |
| --- | --- | --- | --- | --- | --- |
| July 2012 | POLA | 1.65E+06 | 2.66E+07 | 5.84E+04 | 1.64E+05 |
|  | SPOT | 1.62E+06 | 2.41E+07 | 6.56E+04 | 1.23E+06 |
|  | CAT | 4.48E+06 | 4.17E+07 | 2.77E+05 | 2.34E+06 |
| October 2012 | POLA | 1.40E+06 | 2.99E+07 | 9.23E+04 | 1.31E+06 |
|  | SPOT | 1.36E+06 | 2.58E+07 | 1.72E+05 | 1.41E+06 |
|  | CAT | 4.28E+06 | 5.73E+07 | 2.15E+05 | 1.60E+06 |
| January 2013 | POLA | 2.57E+06 | 3.12E+07 | 2.66E+05 | 1.97E+06 |
|  | SPOT | 2.67E+06 | 2.37E+07 | 1.48E+05 | 1.23E+05 |
|  | CAT | 2.70E+06 | 3.21E+07 | 1.72E+05 | 3.08E+06 |
| April 2013 | POLA | 1.67E+06 | 3.22E+07 | 2.29E+05 | 9.23E+05 |
|  | SPOT | 1.28E+06 | 3.94E+07 | 4.67E+04 | 1.54E+06 |
|  | CAT | 2.72E+06 | 5.62E+07 | 8.36E+04 | 5.66E+06 |

| Date | Site | Heterotrophic production (cells/ml/day) | Standard deviation (N=3) |
| --- | --- | --- | --- |
| July 2012 | POLA | 1.95E+06 | 2.54E+05 |
|  | SPOT | 2.96E+05 | 3.11E+04 |
|  | CAT | 5.60E+05 | 5.31E+04 |
| October 2012 | POLA | no data |  |
|  | SPOT | no data |  |
|  | CAT | no data |  |
| January 2013 | POLA | 9.29E+05 | 5.06E+04 |
|  | SPOT | 5.52E+05 | 1.01E+04 |
|  | CAT | 5.29E+05 | 9.01E+03 |
| April 2013 | POLA | 2.28E+06 | 1.79E+05 |
|  | SPOT | 5.77E+05 | 4.82E+04 |
|  | CAT | 6.93E+05 | 1.05E+04 |
