# Supplementary material for: Proteorhodopsins dominate the expression of phototrophic mechanisms in seasonal and dynamic marine picoplankton communities
Source: PeerJ. 2018 Oct 23;6:e5798. doi: 10.7717/peerj.5798 (PMC6202958; doi:10.7717/peerj.5798)
Supplement: Table S2 — See data availability section for raw data accession numbers. [file peerj-06-5798-s002.docx]

|  | Jul-12 | | | Oct-12 | | | Jan-13 | | | Apr-13 | | |
| --- | --- | --- | --- | --- | --- | --- | --- | --- | --- | --- | --- | --- |
|  | CAT | SPOT | POLA | CAT | SPOT | POLA | CAT | SPOT | POLA | CAT | SPOT | POLA |
| total MG read pairs per sample | 37068781 | 31227906 | 37233030 | 28612809 | 18576509 | 10417378 | 31260251 | 35263042 | 55719175 | 7749075 | 34969549 | 48201276 |
| total MT read pairs per sample | 7540004 | 9278629 | 9747496 | 10111631 | 9940196 | 7593272 | 6286578 | 9646814 | 4085213 | 7944665 | 7878167 | 2988218 |
