# Supplementary material for: Proteorhodopsins dominate the expression of phototrophic mechanisms in seasonal and dynamic marine picoplankton communities
Source: PeerJ. 2018 Oct 23;6:e5798. doi: 10.7717/peerj.5798 (PMC6202958; doi:10.7717/peerj.5798)
Supplement: Table S3 — The number of reads (pooled from all samples) that mapped to the assembled ORFs from metatranscriptomes (MT) and metagenomes (MG) was significant compared to number of pooled reads mapped to the curated proteins from MicRhoDE. [file peerj-06-5798-s003.docx]

| **Cluster** | **Number of assembled sequences** | **MG reads mapped to assembled** | **MG reads mapped to curated clade** | **MT reads mapped to assembled** | **MT reads mapped to curated clade** |
| --- | --- | --- | --- | --- | --- |
| Unresolved Proteobacteria | 20 | 2402 | 2639 | 4293 | 4105 |
| CFB | 14 | 1770 | 3332 | 2119 | 9226 |
| Viral | 13 | 1253 | 359 | 1005 | 1996 |
| Gammaproteobacteria | 13 | 1441 | 9566 | 2212 | 43253 |
| Environmental-unknown | 9 | 772 | 4516 | 466 | 24398 |
| Alphaproteobacteria (no SAR11) | 7 | 72 | 10107 | 5 | 40664 |
| Firmicutes | 2 | 69 | 293 | 20 | 3569 |
| Planctomycetes | 2 | 159 | 286 | 509 | 206 |
| Euryarchaea | 2 | 39 | 311 | 0 | 1728 |
| Other rhodopsin | 2 | 160 | 439 | 32 | 365 |
| Halobacteriaceae | 1 | 9 | 14 | 101 | 227 |
| Deltaproteobacteria | 1 | 5 | 47 | 621 | 89 |
