# Supplementary figures and images for: Proteorhodopsins dominate the expression of phototrophic mechanisms in seasonal and dynamic marine picoplankton communities
Source: PeerJ. 2018 Oct 23;6:e5798. doi: 10.7717/peerj.5798 (PMC6202958; doi:10.7717/peerj.5798)

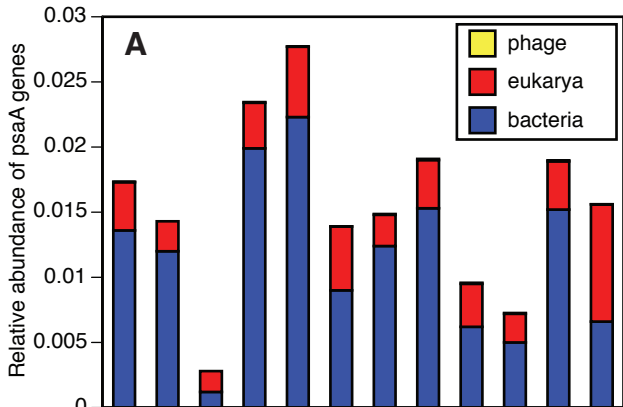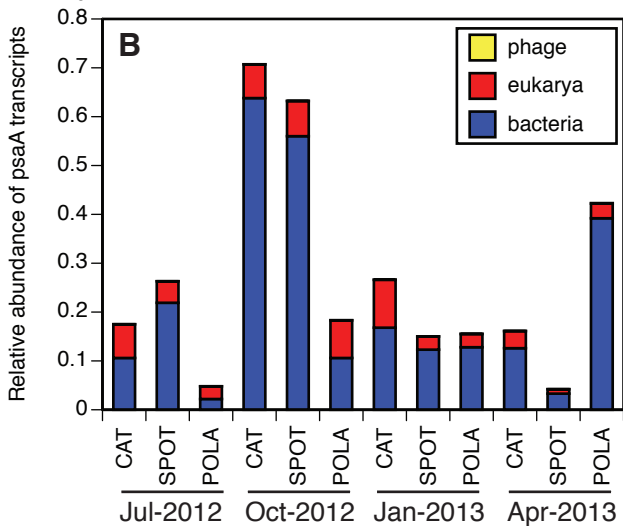

Supplement: Figure S1 — (A) relative gene abundance of psaA by domain (B) relative transcript abundance of psaA by domain. [file peerj-06-5798-s004.pdf]

- Assembled ORFs

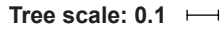

Supplement: Figure S2 — Bacterial sequences appear in blue, viral in red, eukaryotic in green and assembled ORFs in black. [file peerj-06-5798-s005.pdf]

■ pufM

■ psbA

Tree scale: 1 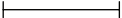

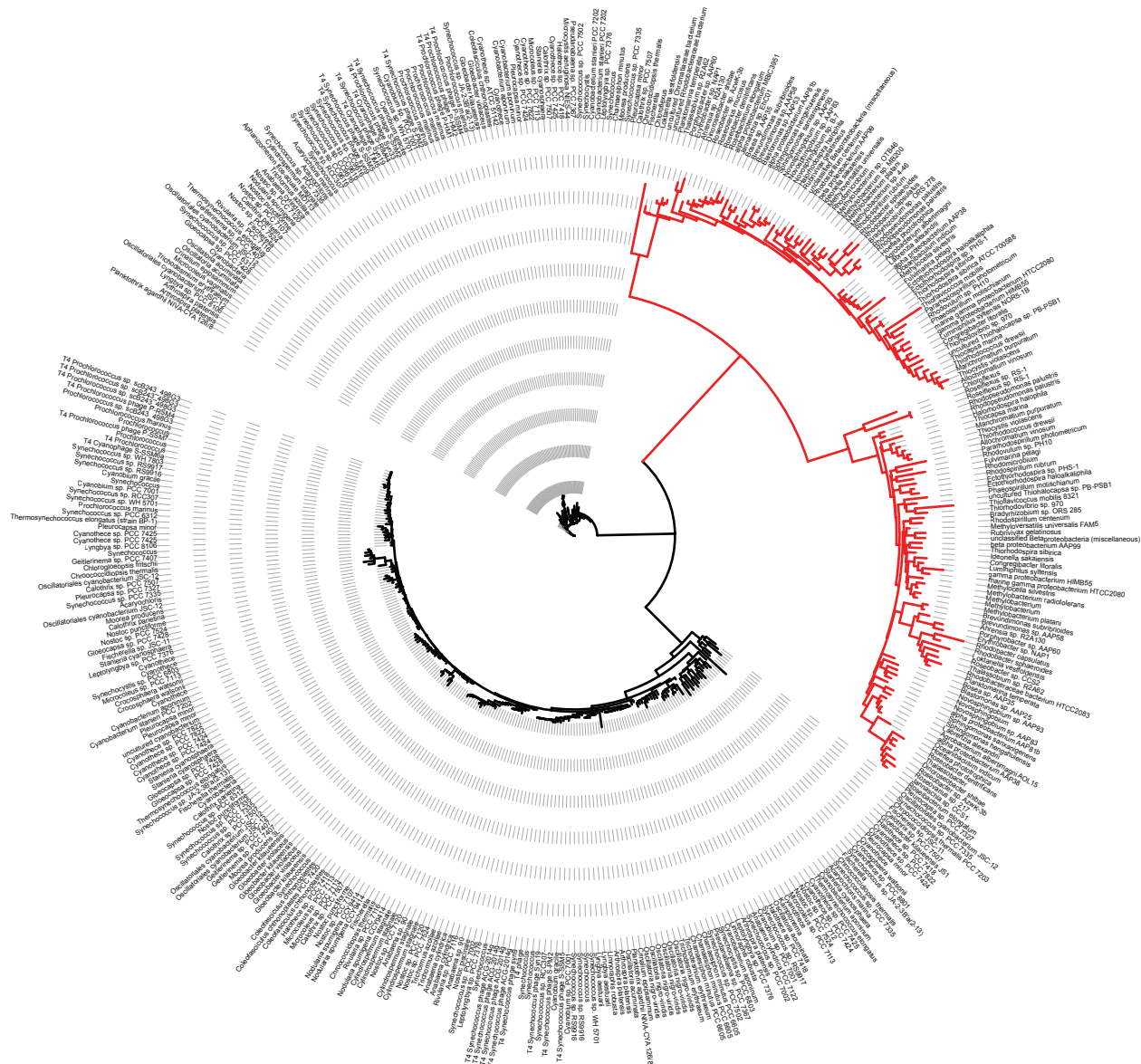

Supplement: Figure S3 — PufM sequences appear in red and PsbA sequences in black. [file peerj-06-5798-s006.pdf]

**A**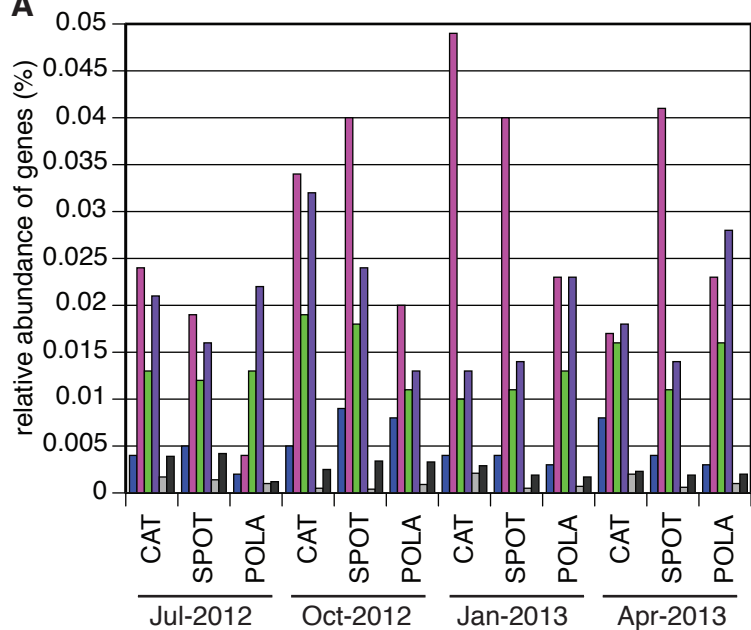**B**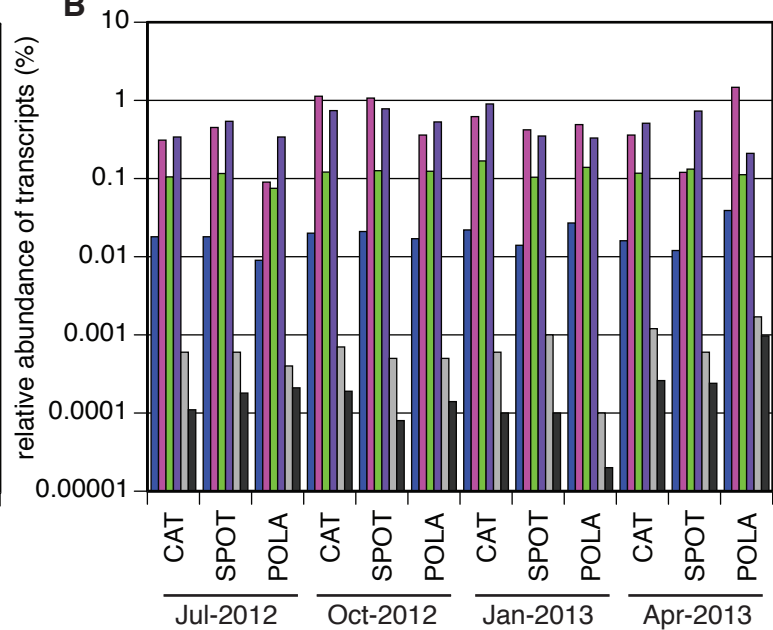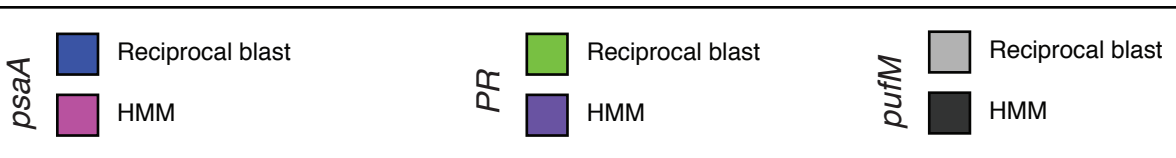

Supplement: Figure S4 — (A) in metagenomes and (B) in metatranscriptomes. Note that the Y-axis in B is logarithmic. [file peerj-06-5798-s007.pdf]

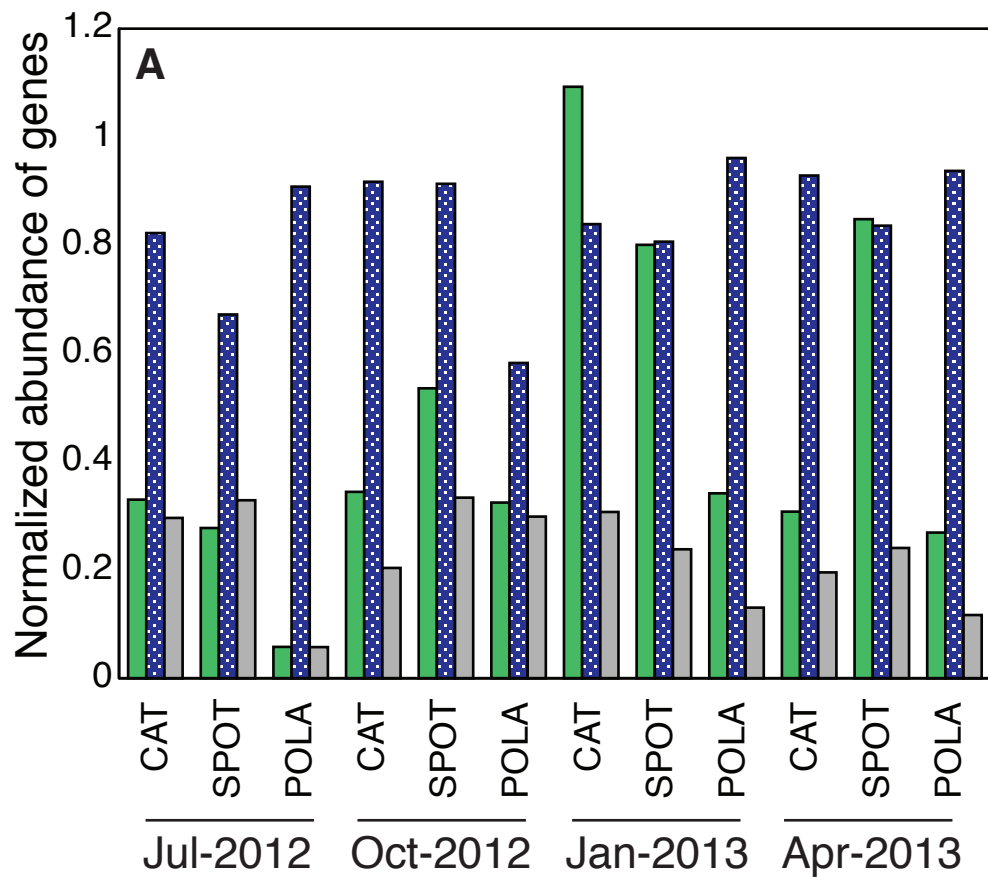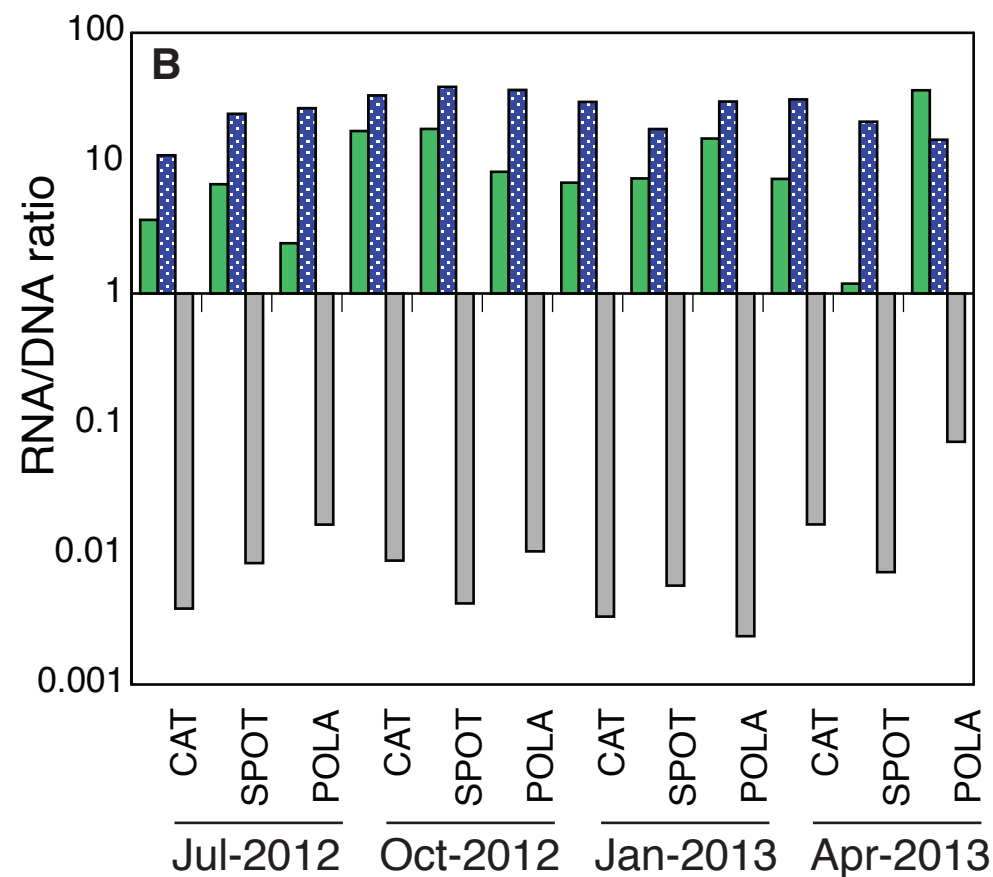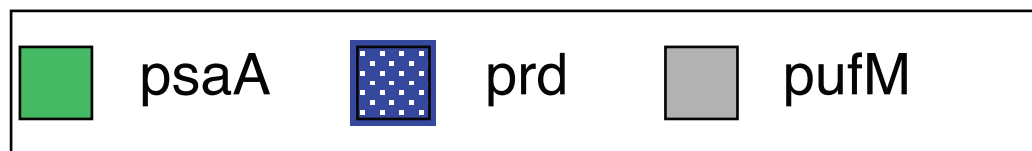

Supplement: Figure S5 — (A) Normalized gene abundance and (B) expression of oxygenic photosynthesis (psaA, green), rhodopsin (PR, dotted blue) and anoxygenic photosynthesis (AAnP, grey). [file peerj-06-5798-s008.pdf]

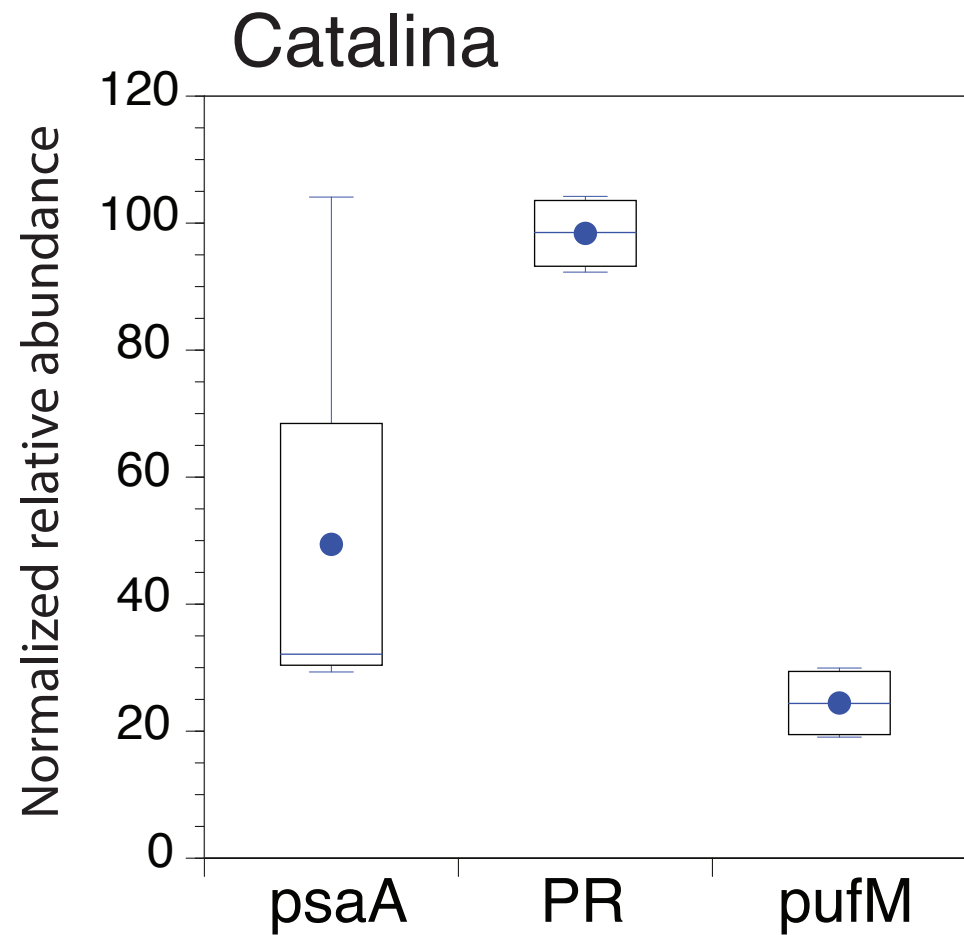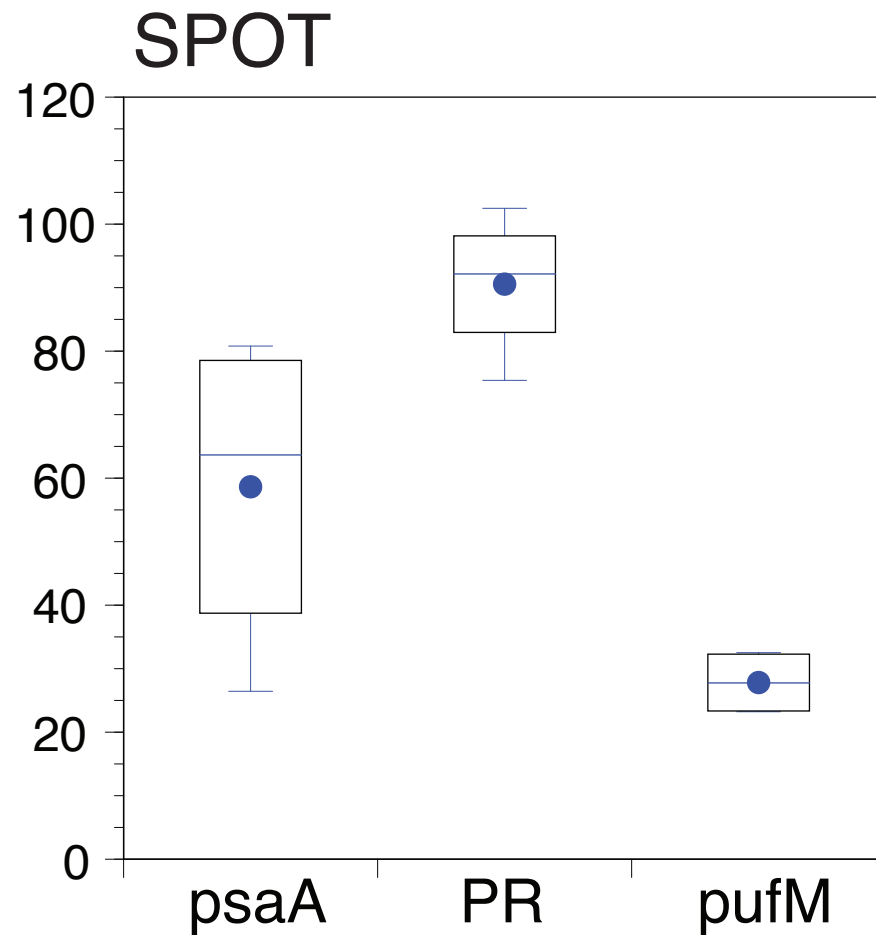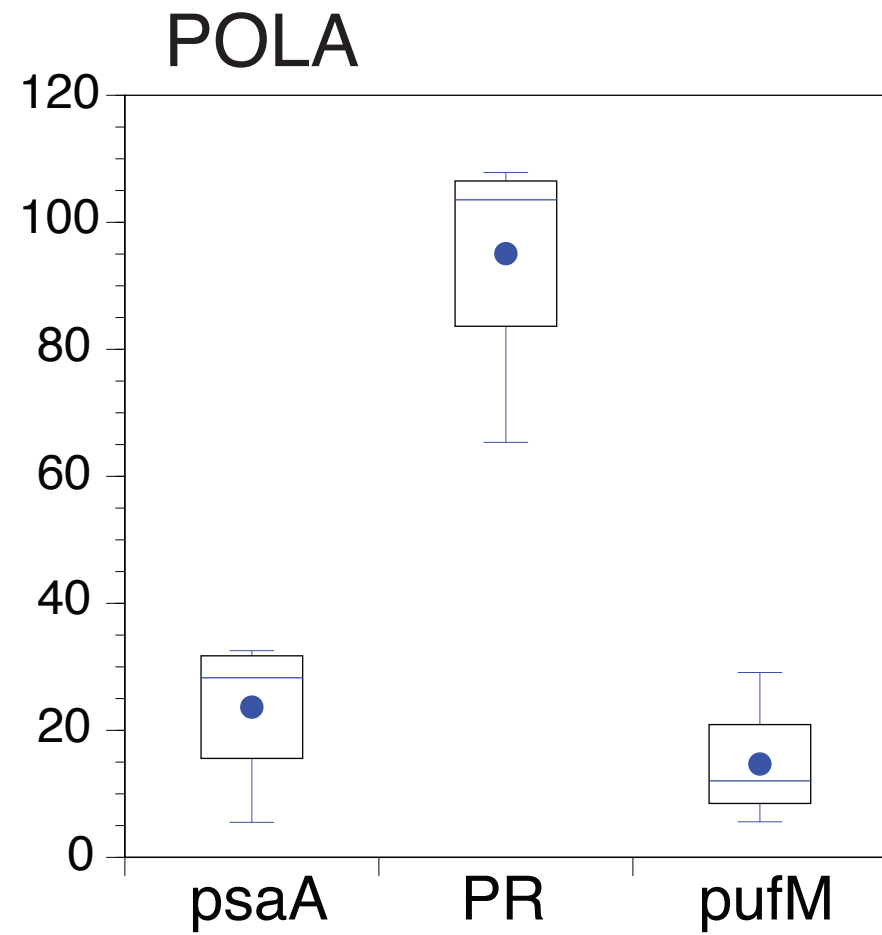

Supplement: Figure S6 — Y-axis denotes the percentage of genomes with the particular phototrophic mechanism normalized to recA gene (see methods). psaA for oxygenic photosynthesis, PR for proteorhodopsin and pufM for aerobic anoxygenic photosynthesis. [file peerj-06-5798-s009.pdf]

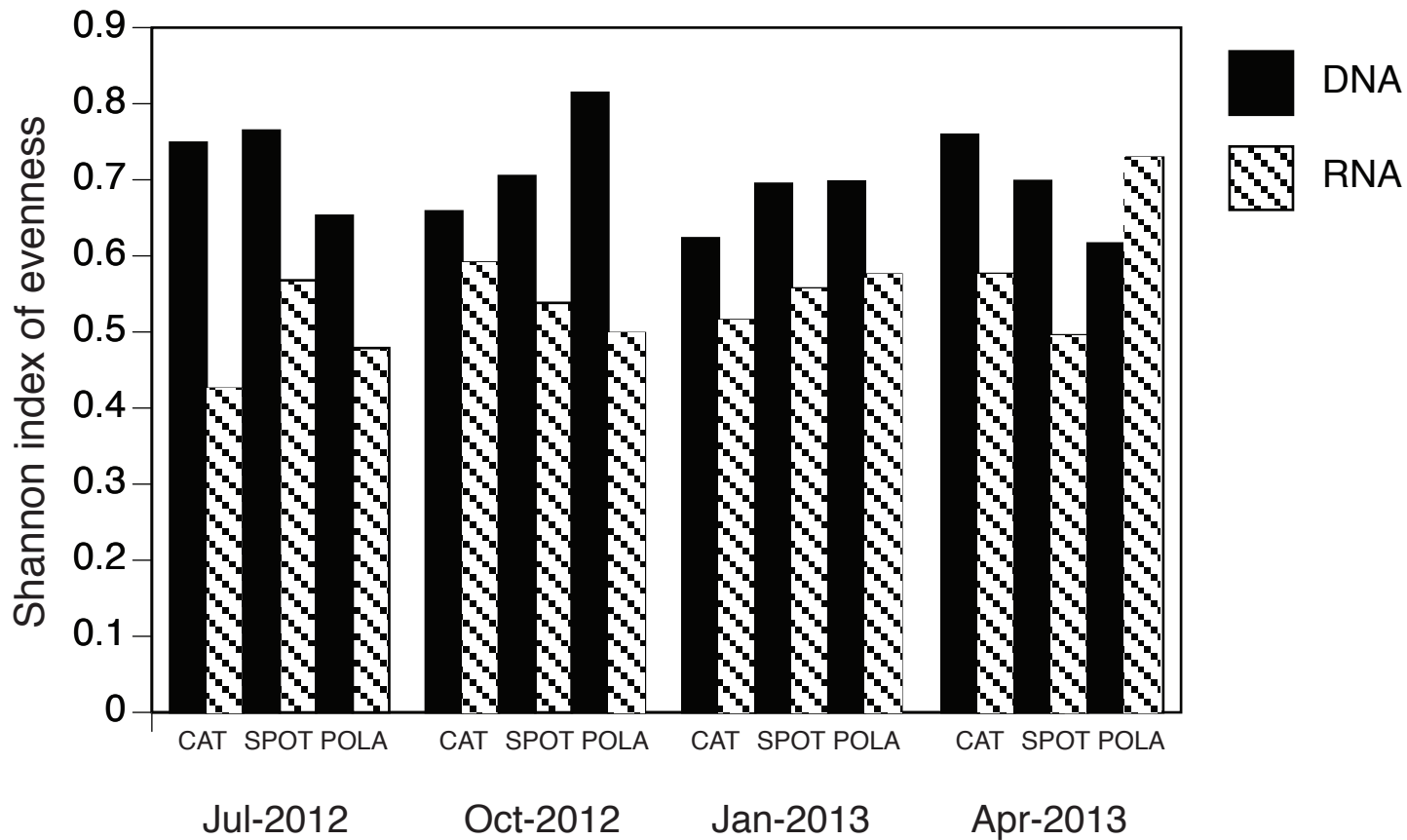

Supplement: Figure S7 — Evenness in metagenomes is denoted by black bars, and in metatranscriptomes by striped bars. [file peerj-06-5798-s010.pdf]

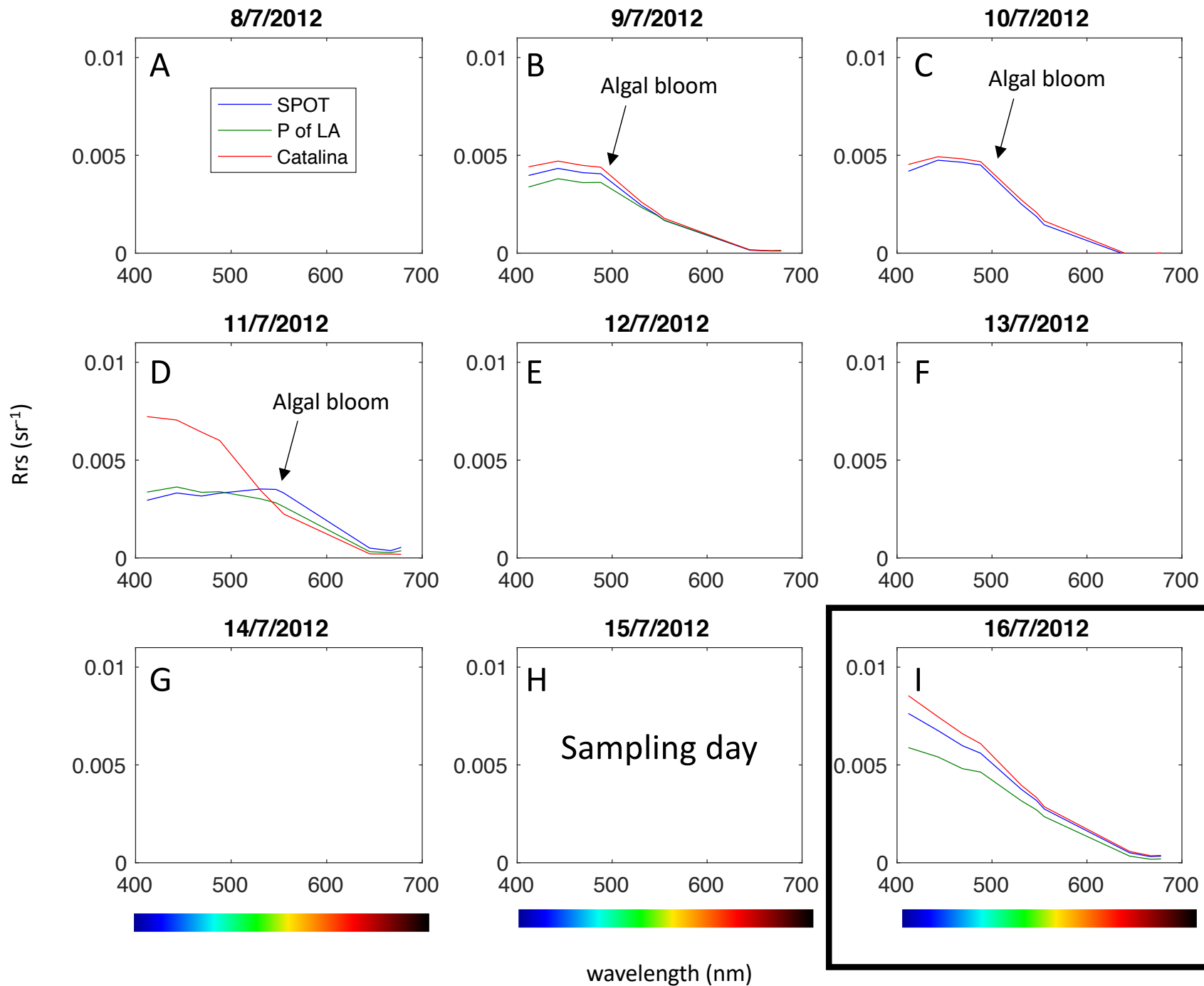

Supplement: Figure S8 [file peerj-06-5798-s011.pdf]
